# Impact of refractive index increment on the determination of molecular weight of hyaluronic acid by muti-angle laser light-scattering technique

**DOI:** 10.1038/s41598-020-58992-7

**Published:** 2020-02-05

**Authors:** Ying Han, Dejie Li, Deqiang Li, Wenwen Chen, Shu’e Mu, Yuqin Chen, Jinling Chai

**Affiliations:** 1 College of Chemistry, Chemical Engineering and Materials Science, Collaborative Innovation Center of Functionalized Probes for Chemical Imaging in Universities of Shandong, Key Laboratory of Molecular and Nano Probes, Ministry of Education, Shandong Provincial Key Laboratory of Clean Production of Fine Chemicals, Shandong Normal University, Jinan, 250014 P.R. China; 2 Center of Research and Development, Bloomage Biotechnology Corporation Limited, Jinan, 250100 P.R. China

**Keywords:** Bioanalytical chemistry, Lasers, LEDs and light sources

## Abstract

Hyaluronic acid (HA) is applied in a number of medical applications and HA of different molecular weight (Mw) are used in different pharmaceutical preparations. In determination of Mw by muti-angle laser light-scattering (MALS), refractive index increment (dn/dc) is an important parameter for accuracy. Herein, the influence of dn/dc on the Mw of HA in stroke-physiological saline solution is investigated by MALS in this work. Additionally, the Mw variation of HA in the manufacturing process of preparations is measured. It is shown that each HA sample corresponds to a specific value of dn/dc, which is varied from 1.38 to 1.74 L/g with the Mw increasing from 13.5 to 2840 kDa in solution. It is indicated by the results from both MALS approach and viscometry that appropriate dn/dc should be selected for Mw determination. In steam sterilization process of preparations at 121 °C, the Mw and conformation of HA can be accurately and rapidly determined by MALS. This work provides a precise method to determine the Mw of HA in the medical applications and preparation industries.

## Introduction

Hyaluronic acid (HA), otherwise known as hyaluronan or sodium hyaluronate, is naturally abundant in mammalian tissues^1^. HA is a naturally occurring linear polysaccharide constituted by repeating units of *N*-acetyl-D-glucosamine and D-glucuronic acid with the monosaccharides linked together by alternating β-1,3 and β-1,4 glycosidic bonds^2^. In nature, HA is found in a wide range of molecular weight (Mw), typically from 10 to 3 × 10^3^ kDa; in solution, chains of HA adopt random-coil conformations^3^. In these conditions, HA is highly hydrophilic and surrounded by water molecules linked through hydrogen bonds. Due to this conformational features, the solutions of this polysaccharide are very viscous and elastic^4^ and their presence in pharmaceutical formulations are characterized by specific Mw segments^5^.

An accurate Mw is a critical factor in the medical application of HA, especially in tumor therapy^6^. Nowadays, HA with different Mw is increasingly utilized for a number of medical applications. Calciu-Rusu *et al*. have investigated the rheology of ophthalmic viscosurgical devices used in cataract surgery and the experimental results were compared with commercial products^7^, the Mw of which is 1.40 × 10^3^ kDa in average. Yeom indicated that a novel, biocompatible, and nontoxic dermal filler was successfully developed with HA of 234 kDa for tissue augmentation^8^. Huh recognized HA of 1.5 × 10^3^ kDa can be effectively used in the mucous membrane spray therapy^9^. Especially, different Mw of HA have different effects on tumor cells. Temieer *et al*. showed that only small molecule HA (less than 5.0 kDa) could induce dendritic cells to mature and promote their production of interleukin and tumor necrosis factor, while HA with high Mw (more than 80 kDa) had no such effect^10,11^.

Based on clinical results, HA injection (1.0 × 10^3^ kDa in average) is an effective drug to treat the osteoarthritis^12^. Although it has been widely used, there are limited number of cases of side effects^13^. The pain and sensation of heaviness for a few hours/days are induced by the treatment of HA injections with high Mw^14–17^ (more than 2.0 × 10^3^ kDa). In patients, the low Mw of HA injected is diminished because of the depolymerization of the long polysaccharide chain and the dilution of HA by arthritic synovial effusions^18,19^. The consequent compromise in viscoelasticity is considered to lead to altered joint mechanics, reduced lubrication and further damage to the diseased cartilage^20,21^. Even several factors may contribute to the occurrence of side effects^22,23^, Mw is still a very important factor that affects the feeling and effect of drug use^24^.

In the past, the Mw of HA was mainly determined by viscometry and empirical formula was used to calculate the Mw based on the data obtained^25^. A major drawback is that as the Mw increases, the viscosity of its aqueous solution increases exponentially, which makes the Mw determination by viscometry to be very difficult^26^. When the Mw of HA is lower than the conventional range, the HA solution is of little viscosity, that will lead to an inaccurate result of Mw determined^25^. In addition, because of the complex pre-processing steps, it is not convenient to use this approach to monitor the Mw variation in the production process of HA preparations^27^. Later, Mw studies of HA have included size-exclusion chromatography experiments and the interference of manual operation is reduced to a certain extent^28^. However, size-exclusion chromatography approach is a purely relative measurement method because the chromatographic system must be calibrated with a series of standards known first^29^. If there is any change in the Mw of the purchased standards, the Mw result of the HA sample would be changed, resulting in a deviated result^30^.

Fortunately, recent investigations on biomacromolecules have employed light-scattering devices^31^. That proves advantageous because light-scattering is an extremely sensitive technique for measuring absolute Mw^32^. For example, Ricci *et al*. investigated the influence of Mw of HA by light-scattering device on the nanovector properties^33^; Botha *et al*. analyzed the Mw of hydrophobically modified HA by size-exclusion chromatography and light-scattering combination method^34^. No reference substances are required in the determination.

It is worth noting that the premise of accurate determination of Mw by light-scattering is to select an appropriate parameter, which is refractive index increment (dn/dc, L/g), because HA in specific polymerization corresponds to an exclusive dn/dc parameter^35^. dn/dc, which is the change in refractive index of solutions as a function of solute concentration, is an essential parameter to several analytical techniques that are based on optical measurements^36^. It is necessary to know dn/dc to characterize the Mw, sizes, shapes and the virial coefficients of polymers^37^. According to the determination principle, small change in dn/dc will greatly affect the result of Mw obtained from light-scattering^38^. Until now, for the selection of dn/dc there are two problems: studies mainly use one dn/dc parameter in Mw research for different HA samples or dn/dc is not properly selected for the Mw determination^39–42^. In addition, different dn/dc parameter in all the applied Mw segments of HA have not been reported yet^43,44^.

In this work, based on the advanced light scattering technology, the size-exclusion chromatography equipment and muti-angle laser light-scattering (MALS) detector are combined to characterize the influence of dn/dc on the Mw of HA in stroke-physiological saline solution and further illustrate the Mw difference of HA sterilized by steam in the manufacturing process of preparations. The important dn/dc parameters of 23 different HA samples with increasing degree of polymerization, named HA1 to HA23, have been determined. Mw results obtained from MALS method and viscometry are compared. Besides, according to the formulation of HA preparations, three batches of preparation are manufactured and the Mw variation as well as the conformation of HA in the process of steam sterilization are monitored by MALS method. It is expected that this work could provide a precise method on the Mw determination of HA in the medical applications and the preparation production process.

## Experimental

### Reagents and materials

23 HA samples with different polymerization degree were obtained from Bloomage Biotechnology Corporation Limited (Jinan, China). Sodium chloride (NaCl), monometallic sodium orthophosphate (NaH_2_PO_4_) and disodium hydrogen phosphate (Na_2_HPO_4_) were obtained from Sinopharm Chemical Reagent Corporation Limited (Shanghai, China). All reagents used were at least of analytical grade. NaH_2_PO_4_ and Na_2_HPO_4_ buffer solution was adopted in order to ensure that the ionic strength and pH of the solution were the same as those injection preparations in pharmaceutical applications.

### Instruments

The size-exclusion chromatography system consists of a high performance liquid chromatography pump unit (Agilent 1260, Agilent Corp., USA), an auto-inject unit (Agilent 1260, Agilent Corp., USA) fitted with a 900 µL quantitative loop and the following column TSK-GEL GMPW_XL_ connected in series. Stroke-physiological saline solution was used as the eluent. The eluent was monitored by the MALS system (Wyatt Technology Corp., USA), which consists of a Dawn Heleos II light-scattering detector and an Optilab rEX Refractometer (RI). Chromatographic signals were captured and analyzed on a computer workstation using the dedicated Astra 6.3 software.

### dn/dc determination

In a state of constant agitation, 8.0 g NaCl, 0.38 g NaH_2_PO_4_ and 1.02 g Na_2_HPO_4_ were added into 1000 mL water in a flask until completely dissolved. Then 1.0 g HA added and the flask was shaken to dissolve HA completely. To determine dn/dc of HA samples in the stroke-physiological saline solution, sample solutions were prepared with different concentrations (in mg/mL) as follows: 0.01, 0.02, 0.05, 0.10, 0.20, 0.50 and determined at 658 nm using the MALS system. The solutions were propelled by a peristaltic pump using a flow rate of 2.7 cm^3^/min. All the experiments were performed at 30 °C. Averaged value of three determination results was reported.

### Viscometry

Viscosity measurements were made in the stroke-physiological saline solution at 30 °C using a four-bulb low-shear capillary viscometer, which is the conventional capillary viscometers of the Ubbelohde type. 0.1 g HA (dried substance) was accurately weighed into a 100 mL volumetric flask and dissolved with the stroke-physiological saline solution of a suitable amount. Then the solvent volume was set with the same solvent to 100 mL, as a stock solution. The stock solution was diluted 10 times with the same solvent to the appropriate concentration on the basis of the flow time as a test solution.

The solvent and the test solution were filtered through a G3 sintered-glass filter and the first 10 mL solution was discarded. The flow time of the subsequent filtrate of the solvent (*t*_0_/s) and the test solution (*t*_1_/s) was determined. The test must comply with the requirements of *t*_1_/*t*_0_ = 1.3~1.5 and *t*_0_ >100.

The following formula (1) was used to calculate the intrinsic viscosity^25^:

1 $$[\eta ]=\frac{\sqrt{2(\frac{{t}_{1}}{{t}_{0}}-\,\mathrm{ln}\,\frac{{t}_{1}}{{t}_{0}})}}{c}$$

Where *η* is the intrinsic viscosity (L/g), *c* the concentration of the test solution (g/L), *t*_0_ and *t*_1_ are the flow time of the solvent and the test solution (s), respectively.

The following formula (2) was used to calculate the Mw^25^:

2 $$[\eta ]=3.6\times {10}^{-4}\,{{\rm{Mw}}}^{0.78}$$

### Preparation monitoring

In a state of constant agitation, 0.80 g NaCl, 0.04 g NaH_2_PO_4_ and 0.10 g Na_2_HPO_4_ were added into 100 mL water in a flask until completely dissolved. Then 1.0 g HA was added and the flask was shaken to dissolve the HA completely. Afterwards, 2 mL of the solution was hot sealed in an ampoule. A group of sealed HA solutions was sterilized in the steam sterilizer (Steris Corp., USA) at 121 °C in different duration. After naturally cooled down to room temperature, the sterilized samples were diluted by the stroke-physiological saline solution to make HA test solution of 0.05 mg/mL. Then, the test solution was filtered by 0.22 μm membrane. The test solution of 500 μL was injected into the size-exclusion chromatography system. The Mw and conformation of HA were recorded by the dedicated Astra 6.3 software on a computer workstation.

## Results and Discussion

### dn/dc and Mw of 23 HA samples

**Figure 1:**
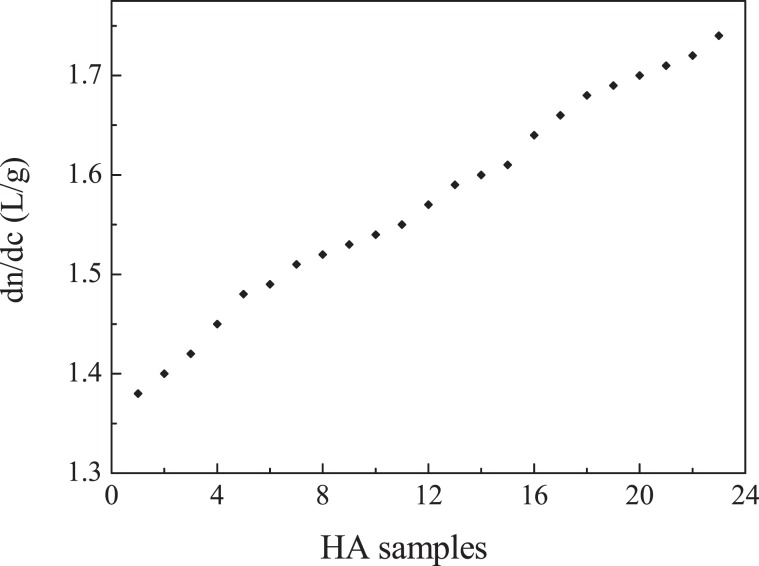
dn/dc profile obtained from 23 HA samples in stroke-physiological saline solution.

23 HA samples propelled by the peristaltic pump using a flow rate of 2.7 cm^3^/min were determined at the temperature of 30 °C. One of the graph of dn/dc determination is shown in Fig. S1 in the Supplementary Information. Limit of detection was calculated as the minimum refractive index change that can be detected over three times the noise standard deviation. The data of dn/dc comes from the average of three determination results. In Fig. 1, it can be found that with the increase of Mw of HA, the value of dn/dc is increasing. For example, HA of 27.6 kDa corresponds to 1.40 L/g and HA of 706 kDa corresponds to 1.54 L/g as the Mw increasing. If the Mw increasing to 2.89 × 10^3^ kDa, the value of dn/dc is further increased to 1.72 L/g. In the experimental result, HA solution sample with Mw of 1.40 × 10^3^ kDa corresponds to the value of 1.60 L/g, which is in good agreement with the value reported in previous research^45^. Baggenstoss carried out the experiments in connection with MALS to make the analysis of HA (500 kDa), obtained a value of 1.53 L/g, which is also the same as our result^46^.

When a polymer with a low degree of polymerization is taken as research object by light scattering at different temperatures, the influence of temperature can not be ignored^47^. The dn/dc of polymer solution may have obvious temperature dependence. Therefore, the influence of different temperatures on the dn/dc parameters of 5 HA samples (HA1, HA6, HA12, HA18 and HA23) are investigated at 20, 30 and 40 °C, respectively. As the result shown in Table 1, maximum difference value of dn/dc in the same polymerization is 0.03 L/g. According to the determination principle of MALS method, this difference is not sufficient to make any deviation to the result of Mw. For example, the Mw of HA1 at 30 °C is 13.5 kDa with the dn/dc of 1.38 L/g and at 40 °C that is also 13.5 kDa with the dn/dc of 1.41 L/g. Even for HA23 which has the largest degree of polymerization among the samples, this Mw difference is just 14 kDa. The deviation is only accounted for 0.6% of the actual Mw. It can be concluded that using the same dn/dc at different determination temperatures such as 20, 30 and 40 °C has little impact on the Mw result.

**Table 1:** The dn/dc (L/g) of HA solution samples at different temperatures.

Samples	Temperatures		
	20 °C	30 °C	40 °C
HA1	1.37	1.38	1.41
HA6	1.49	1.49	1.50
HA12	1.56	1.57	1.58
HA18	1.66	1.68	1.70
HA23	1.72	1.74	1.75

It is worth mentioning that in the published research recently, using one value of dn/dc for a large range of Mw determination is very popular^38–42^. However, different selections of dn/dc can result in different Mw result obtained by the MALS system. The selected experimental data listed in Table 2 proves that it is not appropriate to use just one value of dn/dc to characterize the Mw of HA samples with different polymerization in solutions. For example, the averaged dn/dc of 1.58 L/g is used to calculate the Mw of HA1. If consensus value of 1.58 L/g is adopted, the Mw of HA1 is 12.2 kDa while the actual Mw is 13.5 kDa if the corresponding dn/dc of 1.38 L/g is taken into consideration. The former Mw is 1.3 kDa lower than the actual Mw with the relative deviation of 9.6%. More importantly, as the polymerization of HA increasing, the deviation will be amplified. If dn/dc of 1.38 L/g which belongs to HA1 is used for the determination of HA23, the Mw difference is up to 753.0 kDa and the relative deviation will increase to 26.5%.

**Table 2:** The Mw (kDa) of HA solution samples with different dn/dc (L/g).

Samples	Mw		
	Measured value	dn/dc = 1.58	dn/dc = 1.38
HA1	13.5	12.2	13.5
HA6	213	208	238
HA12	937	937	1.07 × 10^3^
HA18	1.94 × 10^3^	2.10 × 10^3^	2.40 × 10^3^
HA23	2.84 × 10^3^	3.14 × 10^3^	3.59 × 10^3^

### Comparison of Mw obtained by viscometry and MALS

**Figure 2:**
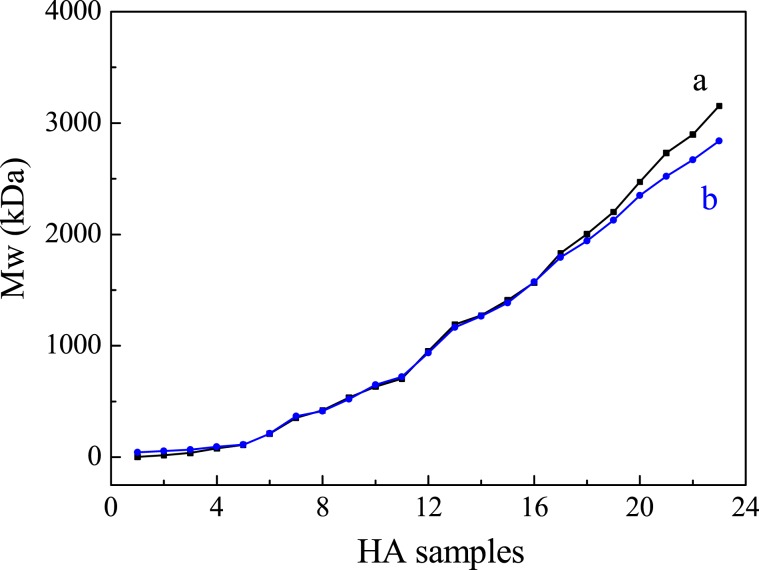
A comparison of the Mw of 23 HA samples obtained from viscometry (**a**) and MALS (**b**).

The Mw of 23 HA samples obtained from two methods including viscometry and MALS are listed in Table S1 and it is more clearly to exhibit the data in a profile. As it is shown in Fig. 2, in the range of 400~1.60 × 10^3^ kDa, the two curves are basically coincident. It is indicated that the Mw obtained by two methods are basically the same within a certain range. The slight difference is at both ends of the curves, where the Mw is less than 400 kDa or more than 1.60 × 10^3^ kDa, the two curves are separated. For the HA sample lower than 400 kDa, the Mw determined by viscometry decreases more than that of MALS; but in contrast, for the HA sample higher than 1.60 × 10^3^ kDa, the Mw determined by viscometry increases more than that of MALS.

The separation is closely related to the nature of HA. In the solution, HA less than 400 kDa is of little viscosity but strong liquidity. The outflow time in the viscometer is so short that the Mw obtained is lower than the real value; on the other hand, HA with high Mw can form a network structure to reduce the fluidity, so the Mw obtained is higher than the real value. Fortunately, MALS combined with the well-known separation technique size-exclusion chromatography, can separate the HA molecular species on the basis of hydrodynamic size and then every fraction of the elution sample can be determined and statistically analyzed. Therefore, MALS is more suitable than viscometry for the determination of Mw and the result obtained from MALS is more reasonable and accurate.

### Mw variation in the sterilization process

**Figure 3:**
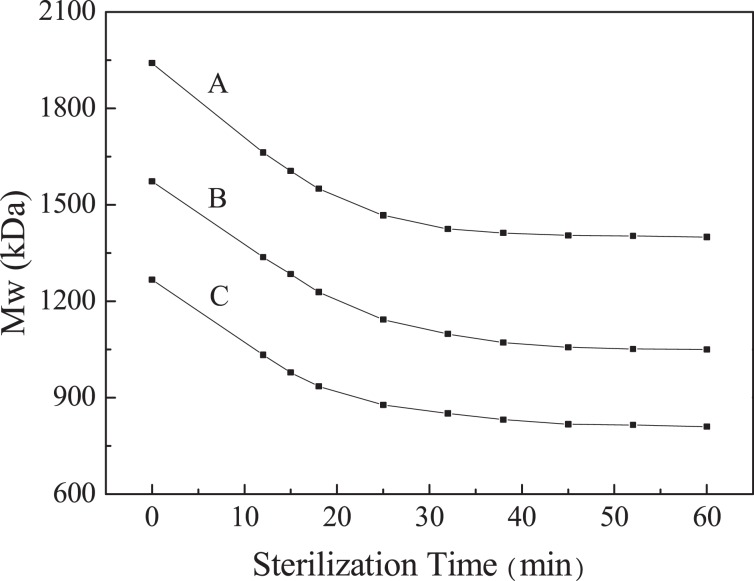
Mw variation of HA samples during the thermal sterilization process.

Control of thermal sterilization treatment for HA solution samples represents a great challenge because the Mw of HA in solution can degrade at a temperature more than 80 °C^48^. Therefore, according to the formulation of HA preparations, three batches of HA solution samples (named A, B and C, respectively) are prepared and the Mw variation is monitored by MALS in the process of sterilization, as depicted in Fig. 3.

The initial Mw of A, B and C are 1.94 × 10^3^, 1.57 × 10^3^ and 1.27 × 10^3^ kDa, respectively. With the time extension, the Mw of HA decrease and they decrease greatly from 0 to 30 min, approximately 30% in average; the decrease rate slows down from 30 to 40 min, only decreases by 20~30 kDa; from 40 to 50 min, the decrease rate is more slower, only decreases by about 10 kDa and the Mw decreases only a little after 50 min. The key point is that when the sterilization time lasting for 40 min, the Mw of sample B will degrade to 1.05 × 10^3^ kDa, which is the optimal Mw for the treatment of osteoarthritis^12^. At that time, the steam sterilization operation should be stopped. The result obtained by MALS method can accurately characterize the Mw variation of HA with the extension of sterilization time.

**Figure 4:**
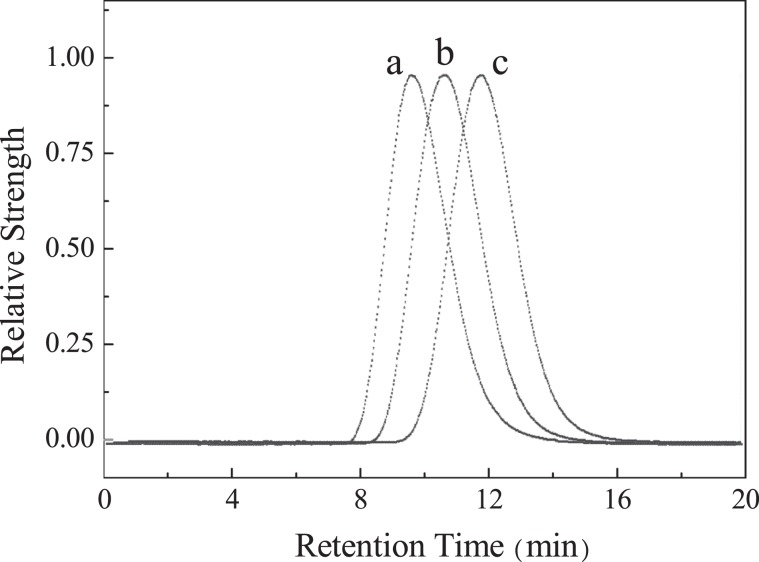
The chromatograms of HA sample before (**a**), during (**b**) and after (**c**) sterilization.

The chromatograms of sample A before, during and after sterilization are shown in Fig. 4. It can be found that all of the fractions of chromatographic peaks move to the right during the sterilization process, showing that every fraction of HA is degraded with the extension of retention time. The Mw variation of sample B and C is in consistent with sample A. All the signals are smooth and no other impurity peaks are found. In addition, the relationship between polydispersity (Mw/Mn) and sterilization duration are listed in Table S2. The greater the Mw/Mn value is, the wider the Mw distribution is, i.e., more dispersed; the smaller the Mw/Mn value is, the narrower the Mw distribution is, i.e., more concentrated. In this experiment, the Mw/Mn values of sample A, B and C before, during and after sterilization are all within 1.10~1.30, which also show good Mw uniformity in the sterilization process. These two facts above indicate that HA degrades uniformly during the sterilization process and no fractions other than HA being generated. Therefore, Mw determined by MALS is not disturbed by the sterilization operation and the result is accurate.

**Figure 5:**
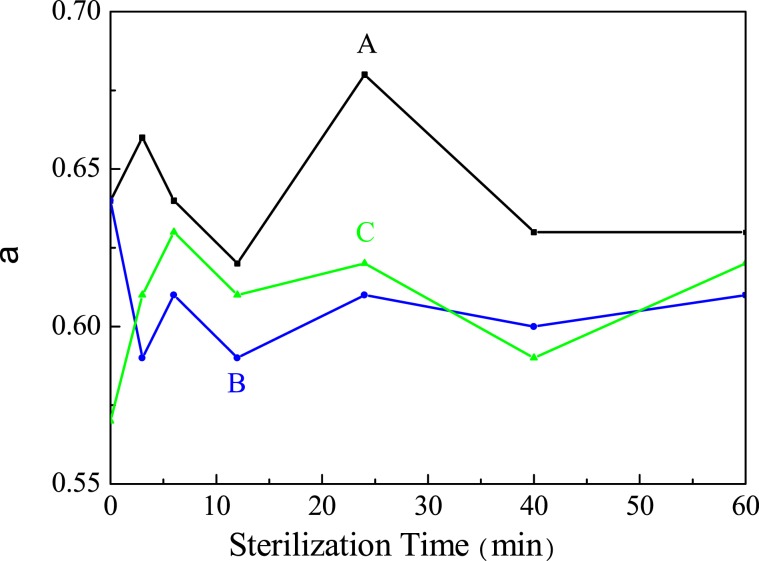
Conformation factor (α) variation of HA samples during the thermal sterilization process.

The detailed conformation factor (α) values of sample A, B and C sterilized at 0, 3, 6, 12, 24, 40, 60 min, respectively, are also listed in Table S2. α value between 0 and 0.4 indicates that HA in solution is in a spherical conformation; α value between 0.5 and 0.8 indicates that HA in solution is in a random coil conformation; α value close to 1.0 indicates that HA in solution is in a rod molecular conformation^49^. According to the profiles shown in Fig. 5, α values of sample A are between 0.62 and 0.68; B are between 0.59 and 0.64; C are between 0.57 and 0.63. All the values are within 0.5~0.7, showing that HA molecules in solution are always in random coil conformation and the steam sterilization operation has little impact on the conformation of HA and also the values of the corresponding Mw determined.

## Conclusions

In this work, the impact of dn/dc on the determination of Mw of 23 HA biopolymer with different polymerization in stroke-physiological saline solution is investigated by MALS method. It is found that with the Mw of HA increasing from 13.5 to 2.84 × 10^3^ kDa, dn/dc varies from 1.38 to 1.74 L/g. Each Mw of HA sample corresponds to a specific value of dn/dc. Comparison of Mw data from MALS and viscometry shows that ranging from 400 kDa to 1.60 × 10^3^ kDa, Mw result obtained by MALS is in good agreement with that of viscometry. It is also indicated that Mw result obtained by MALS with the appropriate dn/dc selected is reliable and accurate. In the steam sterilization operation process, the multiple dispersed HA is degraded and the Mw of this biopolymer is uniformly decreased. Variation of conformation factor indicates that the random coil structure of HA is little influenced by the sterilization operation. Hopefully this investigation work, would be an important step for the understanding of the impact of dn/dc on the determination of Mw by MALS technique in the medical applications of HA and the industrial production processes of preparations.

## Supplementary information

Supplementary Information.
