# Supplementary material for: Impact of refractive index increment on the determination of molecular weight of hyaluronic acid by muti-angle laser light-scattering technique
Source: Sci Rep. 2020 Feb 5;10:1858. doi: 10.1038/s41598-020-58992-7 (PMC7002679; doi:10.1038/s41598-020-58992-7)
Supplement: Supplementary file 1 — Supplementary Information. [file 41598_2020_58992_MOESM1_ESM.doc]

Impact of refractive index increment on the determination of molecular weight of hyaluronic acid by muti-angle laser light-scattering technique

Ying Han, Dejie Li, Deqiang Li, Wenwen Chen, Shu’e Mu, Yuqin Chen, Jinling Chai

Corresponding author.

E-mail address: jlchai@sdnu.edu.cn (J. Chai) chenyuqin@sdnu.edu.cn (Y. Chen)

Figure S1. One of the graphic of dn/dc determined by MALS system.

Table S1. dn/dc and Mw of 23 HA samples obtained from viscometry and MALS.

Table S2. Mw/Mn and α of HA samples in the sterilization process.

Figure S1. One of the graphic of dn/dc determined by MALS system.

Table S1. dn/dc and Mw of 23 HA samples obtained from viscometry and MALS

| Samples | Mw^[a]^  (kDa) | Dn/dc^[b]^  (L/g) | Mw^[c]^  (kDa) |
| --- | --- | --- | --- |
| 1 | 9.01 | 1.38 | 13.5 |
| 2 | 27.6 | 1.40 | 35.7 |
| 3 | 48.8 | 1.42 | 58.2 |
| 4 | 83.1 | 1.45 | 89.2 |
| 5 | 110 | 1.48 | 113 |
| 6 | 212 | 1.49 | 213 |
| 7 | 355 | 1.51 | 369 |
| 8 | 420 | 1.52 | 415 |
| 9 | 535 | 1.53 | 521 |
| 10 | 633 | 1.54 | 650 |
| 11 | 706 | 1.54 | 721 |
| 12 | 954 | 1.57 | 937 |
| 13 | 1.19×10^3^ | 1.59 | 1.17×10^3^ |
| 14 | 1.24×10^3^ | 1.60 | 1.27×10^3^ |
| 15 | 1.41×10^3^ | 1.61 | 1.38×10^3^ |
| 16 | 1.57×10^3^ | 1.64 | 1.57×10^3^ |
| 17 | 1.83×10^3^ | 1.66 | 1.80×10^3^ |
| 18 | 2.01×10^3^ | 1.68 | 1.94×10^3^ |
| 19 | 2.20×10^3^ | 1.69 | 2.13×10^3^ |
| 20 | 2.47×10^3^ | 1.70 | 2.35×10^3^ |
| 21 | 2.83×10^3^ | 1.71 | 2.52×10^3^ |
| 22 | 2.89×10^3^ | 1.72 | 2.67×10^3^ |
| 23 | 3.15×10^3^ | 1.74 | 2.84×10^3^ |

[a] Mw determined by Viscometry. [b] dn/dc determined by MALS system. [c] Mw determined by MALS system.

Table S2. Mw/Mn and α of HA samples in the sterilization process

| Samples | Sterilization duration  (min) | Mw/Mn | α |
| --- | --- | --- | --- |
| A | 0 | 1.16 | 0.64 |
|  | 3 | 1.19 | 0.66 |
|  | 6 | 1.18 | 0.64 |
|  | 12 | 1.20 | 0.62 |
|  | 24 | 1.18 | 0.68 |
|  | 40 | 1.29 | 0.63 |
|  | 60 | 1.19 | 0.63 |
|  |  |  |  |
| B | 0 | 1.18 | 0.64 |
|  | 3 | 1.20 | 0.59 |
|  | 6 | 1.18 | 0.61 |
|  | 12 | 1.21 | 0.59 |
|  | 24 | 1.25 | 0.61 |
|  | 40 | 1.24 | 0.59 |
|  | 60 | 1.21 | 0.61 |
|  |  |  |  |
| C | 0 | 1.20 | 0.57 |
|  | 3 | 1.17 | 0.61 |
|  | 6 | 1.19 | 0.63 |
|  | 12 | 1.19 | 0.61 |
|  | 24 | 1.21 | 0.62 |
|  | 40 | 1.25 | 0.59 |
|  | 60 | 1.24 | 0.61 |
